# Searching for flavor labels in food products: the influence of color-flavor congruence and association strength

**DOI:** 10.3389/fpsyg.2015.00301

**Published:** 2015-03-27

**Authors:** Carlos Velasco, Xiaoang Wan, Klemens Knoeferle, Xi Zhou, Alejandro Salgado-Montejo, Charles Spence

**Affiliations:** ^1^Crossmodal Research Laboratory, Department of Experimental Psychology, University of OxfordOxford, UK; ^2^Tsinghua University, BeijingChina; ^3^Department of Marketing, BI Norwegian Business SchoolOslo, Norway; ^4^Escuela Internacional de Ciencias Económicas y Administrativas, Universidad de La Sabana, ChíaColombia

**Keywords:** flavor words, color, congruence, classification, visual search

## Abstract

Prior research provides robust support for the existence of a number of associations between colors and flavors. In the present study, we examined whether congruent (vs. incongruent) combinations of product packaging colors and flavor labels would facilitate visual search for products labeled with specific flavors. The two experiments reported here document a Stroop-like effect between flavor words and packaging colors. The participants were able to search for packaging flavor labels more rapidly when the color of the packaging was congruent with the flavor label (e.g., red/tomato) than when it was incongruent (e.g., yellow/tomato). In addition, when the packaging color was incongruent, those flavor labels that were more strongly associated with a specific color yielded slower reaction times and more errors (Stroop interference) than those that were less strongly tied to a specific color. Importantly, search efficiency was affected both by color/flavor congruence and association strength. Taken together, these results therefore highlight the role of color congruence and color–word association strength when it comes to searching for specific flavor labels.

## Introduction

According to Garber et al. (2000), most food brands utilize color in one way or another in order to indicate the flavor of the products that they sell. Indeed, when it comes to food, visual cues, together with orthonasal olfaction, are thought to provide most of the information that is available to people before they eat and drink (see also Hutchings, 1977; Stevenson, 2009; Shankar et al., 2010). Color, in particular, may be one of the most important cues guiding people’s flavor expectations, classification, search, and perception (see Stillman, 1993; Garber et al., 2000, 2001; Spence et al., 2010, 2014; Piqueras-Fiszman and Spence, 2011; Velasco et al., 2013).

Flavor information is also often conveyed by means of other design elements on food packaging (Garber et al., 2000). For example, the name of the flavor written on the package, or flavor label, can be important when it comes to deciding which product to buy, since color schemes can vary as a function of brand, product category, and country (e.g., Madden et al., 2000; Piqueras-Fiszman et al., 2012; Velasco et al., 2014). Notably, the congruency (e.g., consistency of the information in terms of consumers’ prior experience) between the elements of marketing communications, such as a product’s packaging flavor label and color, can be critical since congruent (e.g., tomato and red) and incongruent (e.g., tomato and blue) information may have different effects on consumer behavior (Mandler, 1982; Heckler and Childers, 1992; Peracchio and Tybout, 1996; Schoormans and Robben, 1997; Miller and Kahn, 2005).

People match both flavor names (e.g., Piqueras-Fiszman and Spence, 2011; Wan et al., 2014a) and actual flavors (e.g., Zampini et al., 2007; Wan et al., 2014b) to colors in specific ways. The way in which colors and flavors are associated are sensitive to a number of contextual variables (e.g., Shankar et al., 2010). This is presumably attributable to the internalization of the statistical regularities of the environment (Spence, 2011, 2012). Such an idea may explain why it is that certain associations, such as tomato with red, or cucumber with green (natural pairings), are consistent across countries, whereas others are not (the color associated to chicken-flavored crisps can vary across countries, Velasco et al., 2014). A key question here concerns whether color-flavor associations (and the strength of those associations) influence the classification and search for products with specific flavor labels, since the same product/flavor can often be presented in different colors, even in the same country (not to mention new products or colors that may be introduced to the market place).

The literature on the Stroop effect has already demonstrated that when a word (e.g., color name) does not match the (semantically related) color in which the word is presented, people take longer and make more errors when responding to the color of the word (Stroop interference, see MacLeod, 1991, for a review). Importantly, previous studies have demonstrated that the strength of the association between a color and a word can influence the magnitude of interference (Klein, 1964; Scheibe et al., 1967; Proctor, 1978; Klopfer, 1996; Berthet et al., 2011), which may extend to color–flavor word associations. Association strength between color and flavor words is crucial in the context of food products as some flavors may have a stronger color identity (e.g., strawberries) than others (e.g., chicken, see also Tanaka and Presnell, 1999; Lewis et al., 2013, on object color diagnosticity). The strength of the color identity may, in turn depend, for example, on the product category (e.g., an actual chicken has specific colors, which may be different when used in chicken-flavored crisp packaging).

Relevant to the context of the present study, a reversed Stroop effect, in which color interferes with the reading of the word (e.g., Durgin, 2000), has also been documented. Moreover, this effect has been observed in the case of food names and colors. For example, Nijboer et al. (2006) conducted a study in which they assessed color priming in a lexical decision task that used a color patch or word (i.e., a red patch or red word) in order to prime color-related (‘tomato’ and ‘grass’) and color-unrelated words (‘timato’ and ‘griss’), as well as congruent (i.e., red) versus incongruent colors (i.e., blue). In Nijboer et al.’s (2006) study, color facilitated participants’ decisions regarding those words that were semantically related to the color, as compared to incongruent words. In other words, color can influence decision processes and bias them toward related objects/words (see also Heurley et al., 2013). With this in mind, if, say, a flavor label and a color do not match with a consumer’s previous experiences, some kind of interference can be expected when identifying and searching for a product’s flavor information.

A similar color-flavor congruency effect may be expected in the context of visual search for flavor information on a product’s packaging. The way in which people attend to the world around them depends on at least two attentional mechanisms: bottom–up (stimulus-driven) and top–down (goal-driven; e.g., Spence and Driver, 1994, 2004). The latter implies that, by modulating the sensitivity of the brain mechanisms that represent sensory information (top–down processing), it is possible to more efficiently explore and select one kind of stimulus over another (Wolfe and Horowitz, 2004; Knudsen, 2007). People’s goals (e.g., as when searching for a flavor label) can act as a filter that facilitates selective attention to a subset of the visual information (i.e., color) competing for an observer’s attention in the world (e.g., in the supermarket). A particular color-related word or color can activate related representations that may then exert a top–down influence that favors semantically related information. Indeed, knowing (or expecting) the color of a target can reduce search latencies (Theeuwes, 2010; Eckstein, 2011).

The present study builds on Velasco et al.’s (2014) cross-cultural study of color/flavor label associations in crisps packaging. In particular, the flavor words tomato, cucumber, lemon, chicken, and BBQ were used to create both congruent and incongruent stimulus combinations. These flavors were selected because they represented the most frequent color associations across countries, although some variation from one country to another was observed in the case of lemon, chicken, and BBQ. **Table 1** presents a summary of the results of one of the tasks used by Velasco et al. (2014) in which the participants had to select the color that they thought best matched the flavor label. These flavor words were first used in a visual search task (just one set size) and a variant of the go/no-go task. The aim here was to assess whether any Stroop-like effect between packaging colors and flavors was robust. While it may be reasonable to expect such an effect in pairings such as tomato and red, it is, however, less clear whether this would also be the case for previously established associations such as between chicken flavor and the color orange, or between BBQ flavor and a burgundy color (see **Table 1**). Moreover, it is not clear how these associations would influence search efficiency. That said, Experiment 2 was conducted and included a set-size manipulation in order to assess the influence of congruence and association strength (weaker vs. stronger) on search efficiency.

**Table 1:** Most selected colors in one of the tasks used by Velasco et al. (2014), in which the participants had to select the color that they thought best matched each flavor label.

Country	Flavor label	Color
Colombia	BBQ	Burgundy (86.2%)
	Chicken	Orange (77.6%)
	Tomato	Red (96.6%)
	Cucumber	Green (91.4%)
	Lemon	Green (100.0%)
China	BBQ	Burgundy (36.2%)
	Chicken	Orange (44.8%)
	Tomato	Red (75.9%)
	Cucumber	Green (82.8%)
	Lemon	Yellow (65.5%)
UK	BBQ	Burgundy (87.9%)
	Chicken	Orange (69.0%)
	Tomato	Red (93.1%)
	Cucumber	Green (96.6%)
	Lemon	Yellow (100.0%)

The percentages indicate the proportion of participants that selected the color.

## Experiment 1

### Methods and Materials

#### Participants

Thirty-two participants (17 female) aged 18–25 years (*M* = 20.25 years, SD = 1.88) from mainland China took part in this experiment. In the two experiments reported here, all of the participants were Chinese and reported normal or corrected-to-normal vision and no color blindness. They were all recruited from the subject pool of the Applied Cognitive Psychology Laboratory of the Psychology Department at Tsinghua University, Beijing, China. Each participant only took part in one of the experiments, and was either paid (20/25 Chinese Yuan in Experiments 1 and 2, respectively, depending on the duration of the experiment), or else given partial credit to fulfill the requirements of an introductory psychology course in return for taking part in the study. The experiments reported here were all approved by the Central University Research Ethics Committee of the University of Oxford, and were conducted in accordance with the ethical guidelines laid down by the Department of Experimental Psychology at the University of Oxford.

#### Apparatus and Materials

E-Prime 2.0 software (Psychology Software Tools, Inc.) was used to present the stimuli and to collect the data. The participants were seated in front of a 17 inch monitor (at a distance of approximately 50 cm from the screen). The screen had a resolution of 1024 × 768 pixels, and a screen refresh rate of 60 Hz.

**FIGURE 1:**
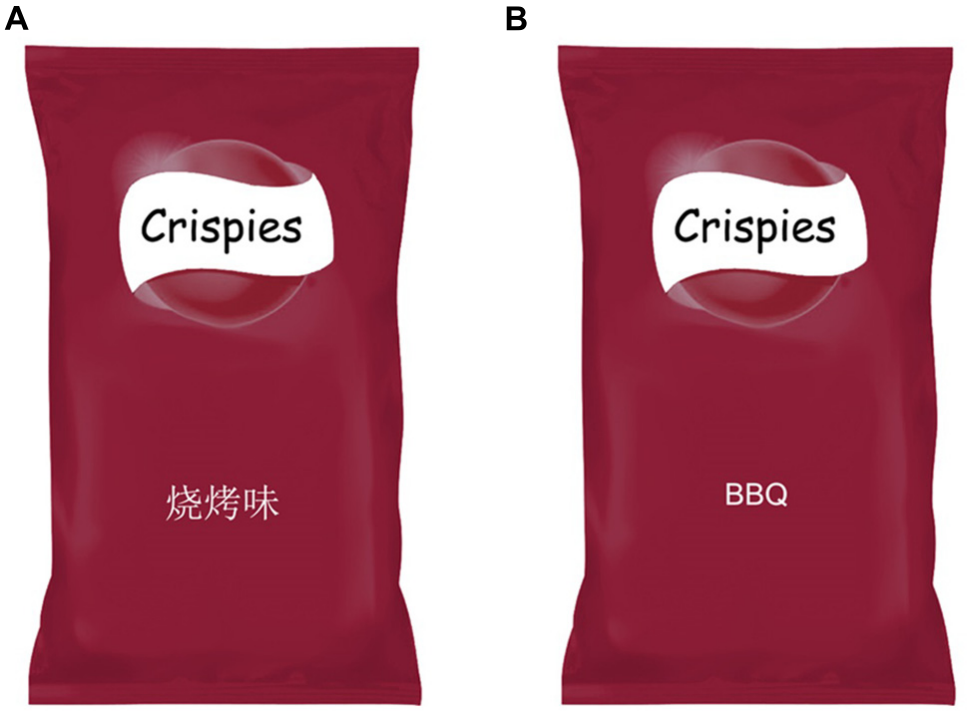
**Example of the packaging used in Experiments 1 and 2. (A)** Shows the flavor written on the package corresponding to BBQ in Chinese. **(B)** Shows the English version.

An image of the packaging of a fictitious brand of crisps (“Crispies”) was modified using Adobe Photoshop CS4 in order to create a set of congruent and incongruent (color/flavor) packaging designs, and additional packaging designs for use in the target-absent trials (see **Figure 1** for an example of the packaging stimuli used in the present study). The packages had a mean luminance of 103.73 Lumas (SD = ±39), and were all presented against a full-screen white background.

The colors that were used were selected from RGB color codes in Adobe Photoshop CS4 as follows: burgundy 8a1d36 (RGB: 138, 29, 54), green 6fae54 (RGB: 111, 174, 84), orange e67f48 (RGB: 230, 127, 72), red d44141 (RGB: 212, 65, 65), and yellow fdd808 (RGB: 253, 216, 8).

The experiment comprised two tasks, a visual search task and a variant of the go/no-go task. The order in which the two tasks were presented was counterbalanced across participants. The targets in both tasks included congruent and incongruent stimuli. The *congruent stimulus combinations* included a total of five packages: BBQ flavor in burgundy, chicken flavor in orange, cucumber flavor in green, lemon flavor in yellow, and tomato flavor in red (based on the results of Velasco et al., 2014). The *incongruent stimulus combinations* included packages with the same flavor labels but now presented in one of the other four (incongruent) colors (i.e., BBQ crisps presented in an orange, green, yellow, or red color). The participants were not told about the congruent or incongruent associations prior to taking part in the study. In addition to the targets, a pool of 20 stimuli was created and used for the no-go and target-absent trials in the go/no-go and the visual search tasks, respectively. This pool comprised four task-irrelevant flavor labels, namely, meat, natural, original, and spice, each of which was presented against the five colors of the targets, namely, burgundy, orange, green, yellow, and tomato.

#### Design and Procedure

##### Visual search task

**FIGURE 2:**
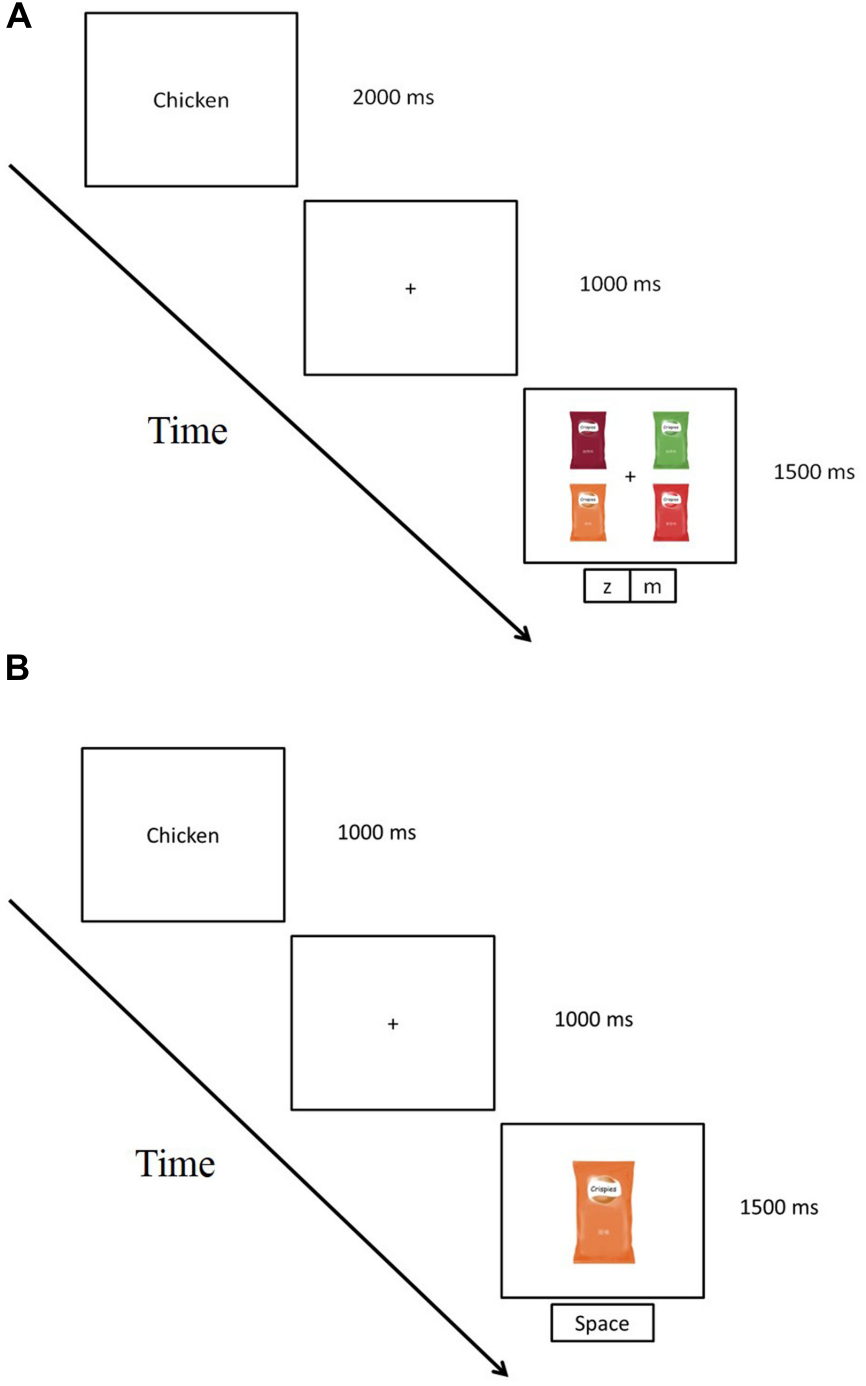
**Schematic representation of a trial in the visual search task **(A)** and a trial in the go/no-go task **(B)** in Experiment 1**.

The participants were presented with written instructions on the computer monitor saying that in every trial, they would first read a target flavor word, followed by a display containing four products, and that their task was to press one key when the target was present and another key when the target was absent. Half of the participants responded by pressing the ‘z’ key for target-present trials, while the other half responded by pressing the ‘m’ key instead (and vice versa for target-absent trials; see **Figure 2A**). All of the participants started the experiment with 15 practice trials, including five congruent, five incongruent, and five target-absent trials, all presented in a random order.

Four packaging designs were presented in every trial in a 2 × 2 array. Three were randomly assigned as distractors and the fourth product was the target (congruent or incongruent in terms of color), or else a non-target product (in target-absent trials). Five packages, selected randomly from the pool of distractors, were used as a list from which one was picked randomly on each target-absent trial. The incongruent packages were randomly selected from a list in which the target flavor was presented in incongruently colored packaging. The positions of the packages were randomized on every trial. Each packaging image was designed to fit within an area of 8.4° × 12.3° of visual angle, and the written flavor names subtended approximately 0.6° × 0.8° of visual angle. The horizontal and vertical distances between the packages consisted of 7.8 and 5.5° of visual angle, respectively. After the practice trials had been completed, the main experiment started. In the main experiment, 75 congruent, 75 incongruent, and 75 target-absent trials were presented randomly, giving rise to a total of 225 trials.

##### Go/No-Go task

At the beginning of the task, written instructions were provided on the computer screen, followed by the practice trials. In each trial, the participants were shown a verbal flavor label (e.g., “tomato,” “chicken,” “cucumber,” “BBQ,” or “lemon,” for 1000 ms), followed by a central fixation cross (for 1000 ms), and then the first packaging image (for a maximum of 1500 ms). Each packaging image was designed to fit within an area of 15.2° (height) × 24.5° (width) of visual angle and the written flavor names subtended approximately 1.5° × 4.3° of visual angle.

The participants’ task (see **Figure 2B**) consisted of pressing the space bar as quickly as possible when the target was presented (either a congruent or incongruent ‘go’ trial) and to withhold any response if a package with a flavor label different from that of the target was presented (‘no-go’ trials). The experiment started with practice trials, including two congruent, two incongruent, and two target-absent trials. In these trials, accuracy feedback was given for 500 ms during the practice trials (the word “correct” following a correct response, “incorrect” following an incorrect response, and “no response was detected” for those trials in which the participants did not make any response). For each incongruent trial, one of the four incongruent packages designed for the targeted flavor was selected at random. No-go trials were also randomly selected from the aforementioned pool of 20 possible target-absent stimuli. After the practice blocks had been finished, the main experiment began. The main experiment comprised two blocks of six congruent, six incongruent, and six target-absent trials for each of the five flavor labels (BBQ, chicken, cucumber, lemon, and tomato), giving rise to a total of 90 trials per block and 180 for the whole experiment.

### Results

#### Visual Search Task

The data from two of the participants were excluded from the analysis due to poor performance (<60% correct)^1^. A paired-samples *t*-test on the error rate revealed that participants made less errors in the congruent (*M* = 12.8% ms, SE = 1.23%) than in the incongruent (*M* = 22.8% ms, SE = 3.0%) condition, *t*(29) = 3.890, *p* = 0.001, Cohen’s *d* = 0.710, 95% CI = -15.2% ≥ μ1–μ2 ≥ -4.7%. From the total of 4500 congruent and incongruent trials including the data from all of the participants, 802 (17.8%) incorrect responses were excluded from the data analysis. In addition, those reaction time (RTs) that fell 2 SDs above or below the mean were excluded from the analysis (resulting in the removal of 2.21% of the remaining trials, meaning that a total of 3616 trials were used in the analyses).

**FIGURE 3:**
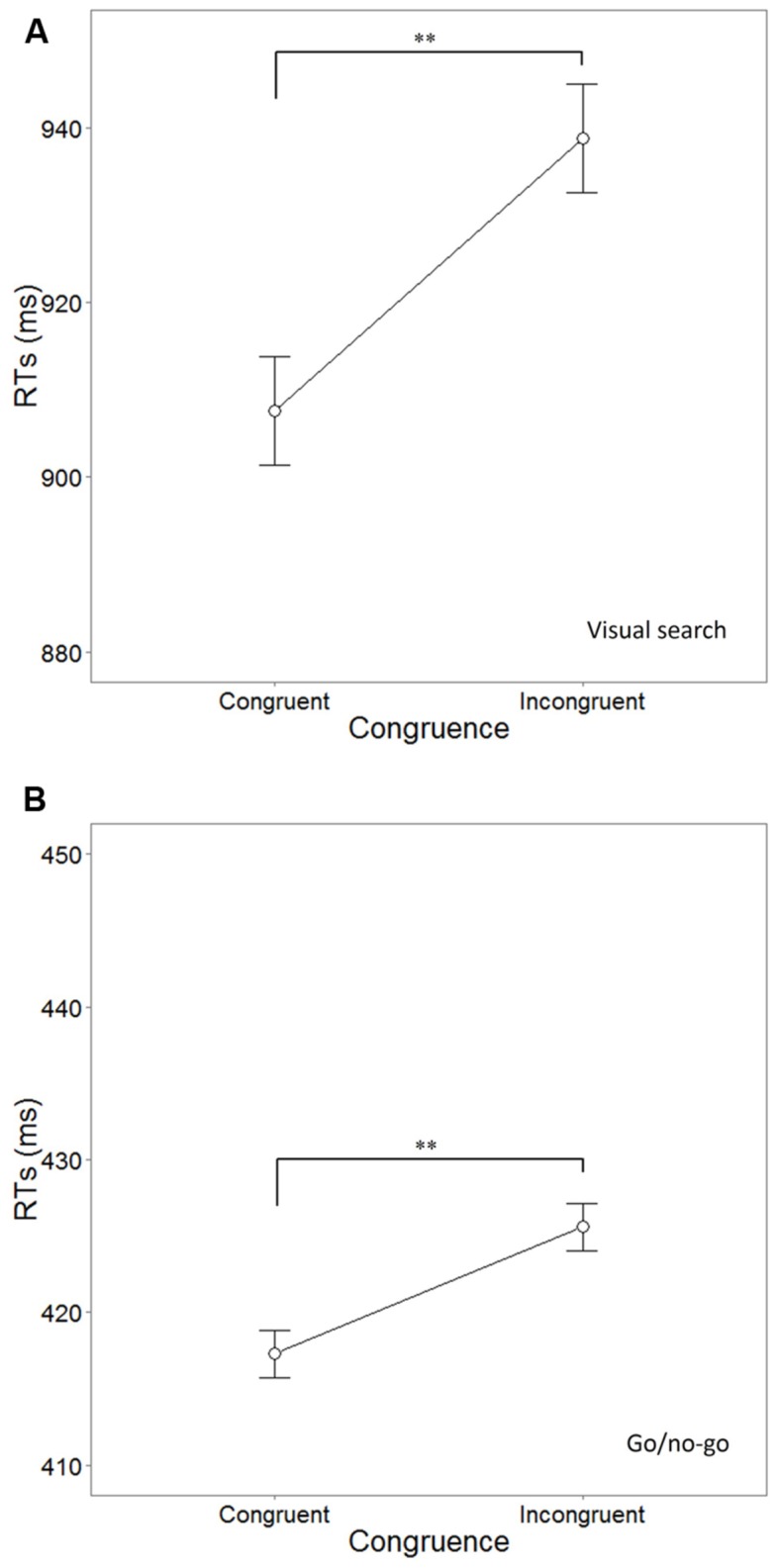
**Mean reaction times (RTs ms) in each congruence condition in the visual search **(A)** and go/no-go task **(B)** tasks in Experiment 1.** The asterisks (^∗∗^*p* ≤ 0.001) highlight the significance of the difference and the error bars represent the SE of the means.

Mean RTs were computed for correct, target-present trials across conditions (congruent and incongruent). A paired-samples *t*-test was used to assess any difference between the congruent (*M* = 908 ms, SE = 15) and incongruent (*M* = 939 ms, SE = 15) trials. This revealed a significant difference of 31 ms, *t*(29) = 3.554, *p* = 0.001, Cohen’s *d* = 0.648, 95% CI = -49 ≥ μ1–μ2 ≥ -13 (see **Figure 3A** for mean RTs). As for the target-absent trials, participants made on average 18.84% (SE = 2.3%) errors, however, no significant difference was found on the error rates between flavor labels in these trials, *F*(4,116) = 0.801, *p* = 0.527, $\eta_{p}^{2}$ = 0.027. On average, the participants responded more slowly to the target-absent trials than the target-present trials (*M* = 1172 ms, SE = 4 ms). No significant difference was found in the target-absent RTs as a function of flavor cue, *F*(4,116) = 2.179, *p* = 0.076, $\eta_{p}^{2}$ = 0.070.

#### Go/No-Go Task

The data from three participants were excluded from the analysis due to their poor performance on the task (<60% correct). From the total of 3480 congruent and incongruent trials including the data from all remaining participants, 76 (2.1%) incorrect responses were excluded from the analysis. A paired-samples *t*-test did not reveal a significant difference between the incongruent and congruent error rates, *t*(28) = 1.077, *p* = 0.29, Cohen’s *d* = 0.237. In addition, those RTs that fell 2 SDs above or below the mean were also excluded from the analysis (4.1% of the 3404 remaining trials, for a total of 3263 used in the analyses). Mean RTs were computed for correct, target-present trials across conditions (congruent and incongruent). The difference in RTs between the congruent (*M* = 417 ms, SE = 10) and incongruent (*M* = 426 ms, SE = 11) trials was significant according to the results of a paired-samples *t*-test, *t*(28) = 3.845, *p* = 0.001, Cohen’s *d* = 0.782, 95% CI = -13 ≥ μ1–μ2 ≥ -4 (see **Figure 3B** for mean RTs). On average, participants made 6.8% (SE = 1.3%) errors in the no-go trials. A repeated measures analysis of variance (ANOVA, Greenhouse–Geisse corrected) did not reveal any difference between the error rates of the no-go trials as a function of flavor cue (“tomato,” “chicken,” “cucumber,” “BBQ,” “lemon”), *F*(3.032,84.905) = 0.333, *p* = 0.855, $\eta_{p}^{2}$ = 0.012.

### Discussion

The results of both tasks provide consistent initial evidence for the idea that color/flavor label congruence facilitates visual search and that, as expected, there is a Stroop-like effect. Participant RTs were slower when responding to flavor words against an incongruent than congruent packaging colors. However, one potential limitation with the interpretation of the results of Experiment 1 relates to the fact that the congruent stimuli always consisted of the same five color/flavor combinations, while the incongruent stimuli were randomly selected from a pool of various incongruent stimuli. One may therefore wonder whether the obtained congruency effects would at least partially be attributable to the differing variety of targets in the congruent versus incongruent trials, and thus the frequency of the same congruent targets (cf. Wolfe et al., 2007; Godwin et al., 2014). Perhaps, less variety in the congruent trials might, in turn, have led to higher familiarity of the congruent stimuli and therefore faster RTs. In order to rule out this alternative explanation, Experiment 2 was conducted. Here, the variety of congruent and incongruent targets was kept the same.

In addition, Experiment 2 moved toward a more complex visual search scenario, and directly addressed two questions: (1) Does color/flavor congruence influence search efficiency? (2) Does the strength of the association between a flavor word and a color affect participants’ search times to flavor words as a function of congruency? To this end, the strength of the association between a flavor word and a color, and the number of packages on the screen (set size), were manipulated. The strength of the association (or association strength) included two levels, namely, weaker and stronger associations. The two-level factor “association strength” was included based on Velasco et al.’s (2014) results. One of the levels (stronger) corresponded to those flavor labels (tomato and cucumber) to which Velasco et al.’s (2014) participants consistently selected the same color across three countries (Colombia, China, and UK) and which were highly associated with a color in China, and the other (weak) to those flavor labels (BBQ and Chicken) to which participants’ associations varied as a function of country and which were only associated by some of the participants to a specific color in China (see **Table 1**). Presumably, some flavors have a stronger color identity due to their frequent co-occurrence with a color in the environment (e.g., cucumbers are green). Here, it was hypothesized that not only should an effect of congruency be expected, but also an effect of association strength (e.g., Velasco et al., 2014). In MacLeod ’s (1991, p. 173) words: *“…as the word’s semantic association to the concept of color increases, so does its potential to interfere.”*

## Experiment 2

### Methods and Materials

#### Participants

20 participants (10 female) aged 18–24 years (*M* = 21.25 years, SD = 1.74) took part in this experiment.

#### Apparatus and Materials

The experimental setting of Experiment 2 followed that of Experiment 1. In Experiment 2, however, exactly four pairs of congruent stimuli and four pairs of incongruent stimuli were used. In particular, the congruent combinations included chicken in orange, BBQ in burgundy, tomato in red, and cucumber in green. The incongruent combinations included chicken in burgundy, BBQ in green, tomato in orange, and cucumber in red. Thus, an equal number of color-flavor label pairs were used in the congruent and incongruent conditions in order to control for the differing variety of these pairings in Experiment 1. All packaging colors were set to equal luminance (122 Lumas). The colors’ RGB codes in Adobe Photoshop CS4 were as follows: burgundy 732532 (RGB: 115, 37, 50), green 2d4511 (RGB: 45, 69, 17), orange 8b2800 (RGB: 139, 40, 0), and red 9e1616 (RGB = 158, 22, 22). The distractors were the same as those used in Experiment 1. In addition, the experimental procedure here manipulated the set size of the packaging display, by including three, six, and nine packages in each search array.

#### Design and Procedure

**FIGURE 4:**
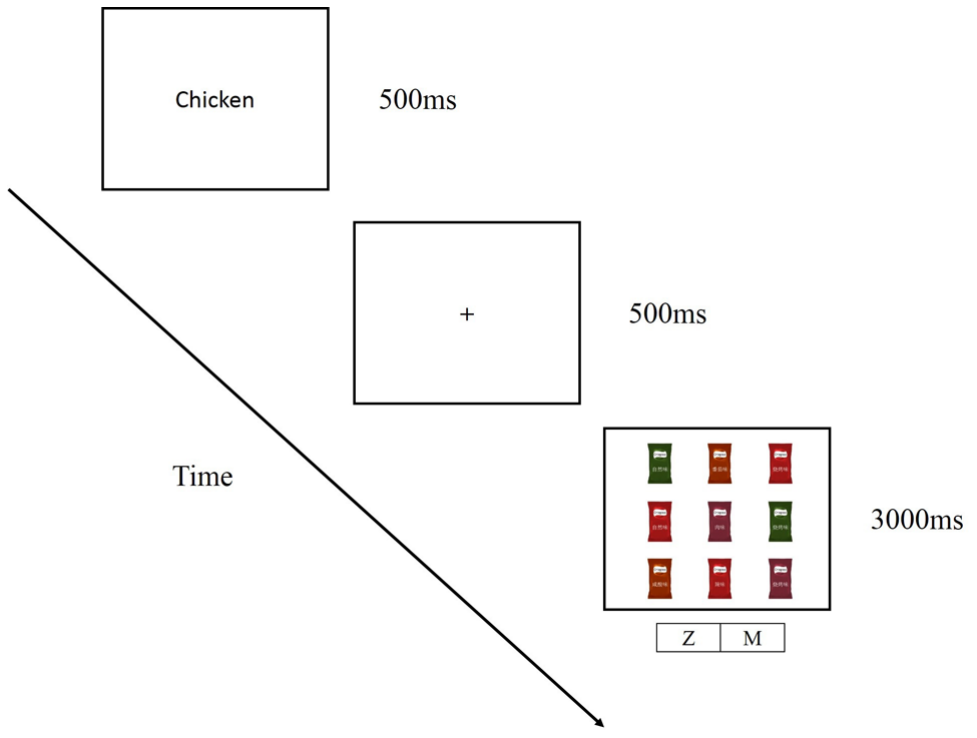
**Schematic representation of a trial in the visual search task (set size = 9) in Experiment 2**.

As in Experiment 1, the participants first completed the practice trials (24 trials). The timing of each trial is illustrated in **Figure 4**. The participants were first presented with a fixation cross for 500 ms, then a verbal flavor label (e.g., “chicken”) for 500 ms, then the fixation cross for 500 ms, and finally the search display with the different packages (3, 6, or 9) for a maximum allowable RT (after which the trial was terminated) of 3000 ms (due to the inclusion of larger set sizes).

After the practice trials, the main experiment started. The design consisted of two blocks of trials. Each block contained 20 congruent, 20 incongruent, and 40 target-absent trials for each set size, giving rise to 240 trials per block, and 480 trials for the whole experiment. Note that 50% of the trials were target-absent trials. When the target was absent, it was replaced by a package that was randomly selected from a list of four packages that were all labeled with “salt and vinegar” but were either orange, burgundy, green, or red. Half of the participants responded by pressing the ‘z’ key for target-present trials, while the other half responded by pressing the ‘m’ key instead. The participants were given a break between the two blocks of trials. In Experiment 2, each packaging image fit within an area of 6.8 × 11.8° of visual angle. The horizontal and vertical distance between the packages was 7.8 and 1.6° of visual angle for the set size with nine packages. The distances between the packages in the other set sizes (three and six) varied as a function of the position of both the target and the distractors, which were randomly assigned to any of the nine locations used in the bigger set size. The flavor names written on each package subtended approximately 1.16° × 2.98° of visual angle.

#### Analyses

We performed two separate 3 (set size: three, six, nine items) × 2 (association strength: weaker, stronger) × 2 (congruence: congruent, incongruent) repeated-measures ANOVAs on both the error rates and RTs, and a 2 (association strength) × 2 (congruence) ANOVA on the slopes that resulted from each condition. In addition, the error rates and RTs of the target-absent trials were analyzed by means of a 3 (set size: three, six, nine) × 2 (association strength: weaker and stronger) ANOVA.

### Results

#### Error Rates

A summary of the error rate data is presented in **Table 2**. Significant main effects of set size, *F*(2,38) = 6.326, *p* = 0.004, $\eta_{p}^{2}$ = 0.250, association strength, *F*(1,19) = 6.774, *p* = 0.017, $\eta_{p}^{2}$ = 0.263, and congruence, *F*(1,19) = 8.552, *p* = 0.009, $\eta_{p}^{2}$ = 0.210, were found. Furthermore, the analyses revealed a significant 2-way interaction between association strength and congruence, *F*(2,38) = 13.327, *p* = 0.002, $\eta_{p}^{2}$ = 0.412. Bonferroni-corrected pairwise comparisons revealed that the participants made more errors in the larger set size (*M* = 13.13%, SE = 1.45%) than in the 3- (*M* = 8.19%, SE = 1.06%) and 6-packaging (*M* = 10.31%, SE = 1.30%) set sizes (*p* = 0.019 and *p* = 0.034, respectively). In addition, the participants made more errors when the target was a flavor label with a stronger color association (*M* = 11.75%, SE = 1.11%) than when it was one of those with a weaker association (*M* = 9.33%, SE = 1.10%, *p* = 0.017). Similarly, the participants made more mistakes in the incongruent (*M* = 12.17%, SE = 1.06) than in the congruent (*M* = 8.92%, SE = 1.23%) condition (*p* = 0.009). As for the interaction term, it was found that, in the incongruent conditions, the participants made more errors when responding to the flavor labels with a stronger color association (*M* = 14.58%, SE = 1.49%), than those with a weaker association (*M* = 9.75%, SE = 1.31%; *p* = 0.004).

**Table 2:** Error rates for both target-present, congruent, and incongruent, trials, as well as target-absent trials, as a function of both association strength and set size.

Congruence	Association strength	Set size (ER%)		
		3	6	9
Congruent	Weaker	6.75	7.00	13.00
	Stronger	6.00	9.75	11.00
Incongruent	Weaker	8.00	9.50	11.75
	Stronger	12.00	15.00	16.75
Target-absent	Weaker	6.38	7.88	11.00
	Stronger	6.75	9.25	10.63

The analysis of the error data (using the Greenhouse–Geisser correction) of the target-absent trials only revealed a significant main effect of set size, *F*(1.529,29.045) = 6.605, *p* = 0.008, $\eta_{p}^{2}$ = 0.258 (see **Table 2**). The participants made fewer errors in the smallest packaging set size (*M* = 6.6%, SE = 2.4%) than in the 9-packaging set size (*M* = 10.8%, SE = 3.1%; *p* = 0.012). A marginally significant difference between the 3- and the 6-packaging (*M* = 8.6%, SE = 2.9%) set sizes was also observed (*p* = 0.058).

#### Reaction Times

**FIGURE 5:**
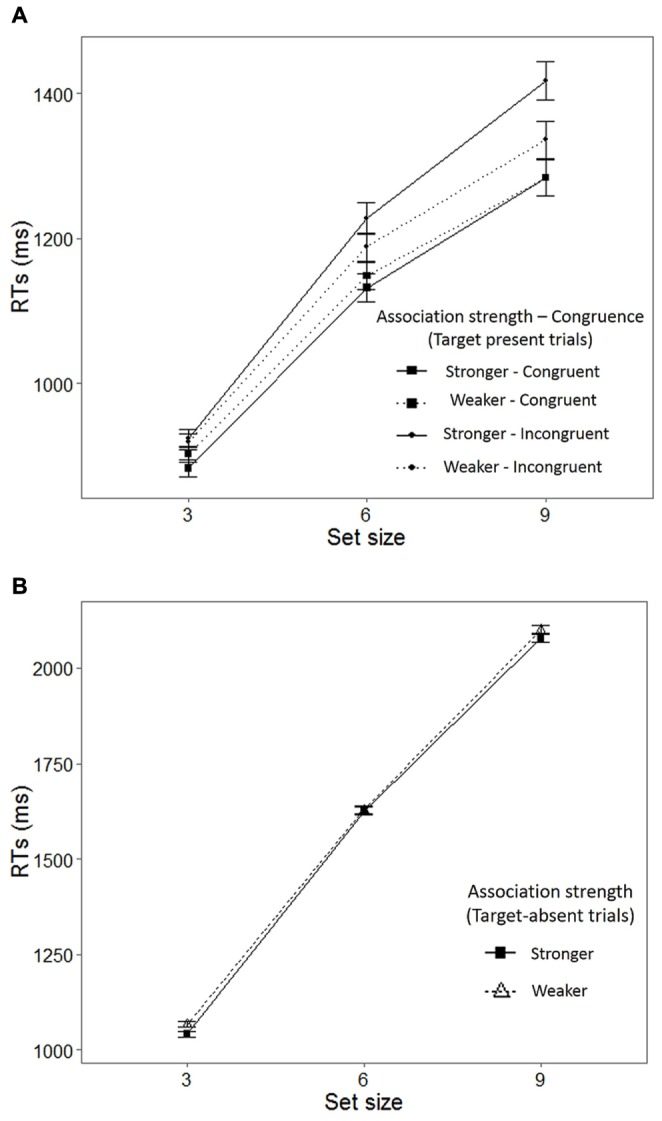
**Summary of the results of the visual search task in Experiment 2. (A)** Mean RTs to target-present trials as a function of set size, association strength, and congruence, and **(B)** Mean RTs to target-absent trials, as a function of set size and association strength. The error bars represent the SE of the means.

From the 4800 congruent and incongruent trials from all of the participants, 506 trials with incorrect responses (10.54%) were excluded from the RT analysis. In addition, those RTs falling 2 SDs either above or below the mean of each set size condition’s RTs were excluded from the data analysis (3.98% of the remaining trials, leaving 4123 trials in the final analyses). The results revealed a significant main effect of set size, *F*(2,38) = 338.946, *p* < 0.001, $\eta_{p}^{2}$ = 0.947, and congruence, *F*(1,19) = 37.073, *p* < 0.001, $\eta_{p}^{2}$ = 0.661, and a significant two-way interaction between association strength and congruence, *F*(1,19) = 5.978, *p* = 0.024, $\eta_{p}^{2}$ = 0.239. In addition, the results also revealed a marginally significant interaction between set size and congruence, *F*(2,38) = 3.195, *p* = 0.052, $\eta_{p}^{2}$ = 0.144. No significant effects were found for association strength, the interaction between set size and association strength, or the interaction between set size, association strength, and congruence (see **Figure 5** for a visualization of the results).

Bonferroni-corrected pairwise comparisons revealed that the participants responded faster to the targets in the set size with three packages (*M* = 910 ms, SE = 21) than to those in the other two set sizes, and more rapidly to the targets in the set size with six packages (*M* = 1179 ms, SE = 30) than to those in the set size with nine packages (*M* = 1339 ms, SE = 30, *p* < 0.001, for all comparisons). Moreover, it was found that the participants responded more rapidly to the congruent (*M* = 1112 ms, SE = 24) than the incongruent trials (*M* = 1174 ms, SE = 28; *p* < 0.001). As for the interaction between association strength and congruence, the analyses revealed that, in the incongruent conditions, the participants responded more rapidly to the flavor labels with a weaker color association (*M* = 1149 ms, SE = 29) than to those with a stronger color association (*M* = 1191 ms, SE = 29; *p =* 0.028).

The analysis of the RT data of the target-absent trials (see **Figure 5B**) only revealed a significant effect of set size (note that the Greenhouse–Geisser correction is used), *F*(1.226, 23.301) = 725.604, *p* < 0.001, $\eta_{p}^{2}$ = 0.974. The participants identified that a target was absent faster in the 3-packaging set size, followed by the 6-, and 9-packaging set sizes (*p* < 0.001, for all comparisons).

Details of the mean RTs, slopes, and intercepts for each condition are shown in **Table 3** (see also **Figure 5**). A two (association strength: weaker and stronger) × 2 (congruence: congruent and incongruent) ANOVA was performed on the slope values. The analysis revealed significant main effects of association strength, *F*(1,19) = 5.925, *p* = 0.025, $\eta_{p}^{2}$ = 0.238, and congruence, *F*(1,19) = 5.052, *p* = 0.037, $\eta_{p}^{2}$ = 0.210. The interaction was not significant. The slopes were steeper when the participants responded to the flavor words with a stronger (*M* = 76, SE = 3) than a weaker (*M* = 67, SE = 4) association strength (*p* = 0.025). Likewise, the slopes were steeper in the incongruent (*M* = 77, SE = 4) than the congruent (*M* = 66, SE = 3) combination (*p* = 0.037).

**Table 3:** Mean reaction times (RTs; in ms) as a function of set size, association strength, and congruence in Experiment 2.

Congruence	Association Strength	Set size (mean RTs)			Slope	Intercept
		3	6	9		
Congruent	Weaker	919	1148	1284	64	729
	Stronger	923	1132	1284	67	697
Incongruent	Weaker	901	1188	1333	70	730
	Stronger	883	1228	1418	83	694
Target-absent	Weaker	1067	1629	2099	172	566
	Stronger	1041	1625	2078	172	544

The last two columns provide the mean slopes and intercepts (all significant, *p* < 0.001).

### Discussion

The results of Experiment 2 therefore replicate those of Experiment 1 while providing further evidence to suggest that the effect is still present across different set sizes. In addition, they support the idea that when the packaging color is incongruent those flavor words to which a color is more strongly associated will be harder to find than those which are less strongly associated to a color. Importantly, search efficiency is affected by both congruence and association strength, with congruent combinations producing more efficient searches on the one hand, and flavor words with a weaker association strength on the other. There are several points to be mentioned here: First, color is a preattentively processed feature that can guide attention more efficiently toward a target, producing near-flat search slopes (Wolfe and Horowitz, 2004). In the context of the present study though, when the color of the packaging is congruent but the word is not the target (mismatch), participants then have to move to another target, thus, producing slower, more effortful, searches (see also Grossberg et al., 1994). Moreover, the strength of the association may also be key as participant’s previous associations between a flavor and a word may then determine which color would guide attention. Finally, while the aforementioned points may help to explain why color does not seem to produce efficient searches, it is also worth mentioning that the packages used as distractors could also have had a color that was semantically congruent with the target (though with a distractor flavor label). Such an element may have increased noise (cf. Wolfe, 2005), and thus made search less efficient as if, say, each search trial had had categorically different colors (target-distractor similarity).

## General Discussion

The two experiments reported in the present study provide evidence that the visual search for product packaging is influenced by color-flavor congruence and association strength. Specifically, participants searched for the target flavor labels more rapidly when they were presented in a packaging having a color that was congruent (vs. incongruent) with the cued flavor. These results are consistent with the idea that color can facilitate participants’ responses toward concepts that are semantically related to colors in a Stroop-like fashion (e.g., MacLeod, 1991; Durgin, 2000; Nijboer et al., 2006; Richter and Zwaan, 2009). It is possible that a particular word can activate object-related representations that may then exert some kind of top–down influence on the tasks. That is, when the actual object fits the participant’s expectations, a facilitation effect can be observed. In addition, incongruent colors tend to interfere more with flavor words that have a stronger color identity (e.g., cucumbers tend to be green) than with those with a weaker color identity (e.g., chicken can be orange in the context of crisps).

The results of the two experiments reported here are also consistent with the literature on the Stroop and the reverse Stroop effects (see MacLeod, 1991, for a review). According to this literature, a color word can influence the way in which people respond to the font color and a physical color can also influence participants’ responses to a color word (e.g., Richter and Zwaan, 2009). A particular word or color can activate related representations that may then exert a top-down influence on the tasks. In other words, the visual elements that people link to a particular food or food word can influence the visual search for food products as a function of congruency.

The interaction between association strength and congruence that was observed in Experiment 2 is comparable to one of the experiments (Experiment 3) reported by Berthet et al. (2011). By using both an affective priming and a Stroop task, they found an interaction between relevant information strength (e.g., how strongly a target is associated with a color) and congruence (valence-picture for the affective priming task and color-object for the Stroop task). In particular, Berthet et al. (2011) documented a significant difference between congruent and incongruent trials in the stronger condition, but not in the weaker one. While in the present study a difference between congruent and incongruent trials was found both in the weaker and stronger condition, it appears as if the difference is larger in the stronger condition than in the weaker one. This, in turn, suggests greater interference, which is reflected in the difference between weaker and stronger conditions in the incongruent trials.

Here it is important to mention that the pattern of performance observed in the target-absent trials in Experiment 2 should be interpreted with some degree of caution. When the target was absent, one of four possible packages, labeled with “salt and vinegar” and either orange, burgundy, green, or red color, was randomly selected and used in the trial. As only one flavor label was used, it is possible that our participants may have adopted a strategy whereby they responded ‘absent’ as soon as they saw ‘salt and vinegar’ (and hence a target), with the second target being the intended target for that trial. However, one finding that suggests that this strategy was not adopted by the participants is the observation that the mean slope of the target-absent trials was more than twice as steep as the slopes of both the congruent and incongruent target present trials. This slope pattern is typically observed for target-absent versus target-present trials (cf. Wolfe, 1998). In addition, the results regarding the main hypothesis of this study would be unaffected by such a response strategy; and the main hypothesis is therefore still supported by the results of both experiments.

The results reported here therefore highlight the importance of flavor labeling and color congruence when it comes to classifying and searching for flavor information. As suggested by Velasco et al. (2014), it is likely that the presented color/flavor label associations are learnt by internalizing the statistical regularities in the environment (i.e., common pairings between colors and flavors). Such regularities also extend to the market place (e.g., Labrecque and Milne, 2013), and frequently, object/color co-occurrences can be internalized by the consumer and subsequently influence information processing as a function of the level of congruency (e.g., Clydesdale, 1991; Piqueras-Fiszman et al., 2012; Velasco et al., 2014). The experimental approach outlined here may be useful for those marketing practitioners interested in launching a new product, or those thinking about changing the color of their current product offerings. By assessing the effects of specific color-related words such as flavor words, marketers may be able to understand which colors may or may not interfere with such words.

The Stroop-like effect observed here is also compatible with the literature on visual search. Eimer (2014) recently put forward a model of selective attention designed to account for the existence of four key stages in visual search, which include preparation, guidance, selection, and finally identification. Preparation refers to the activation of representations, or ‘target templates’ in working memory, related to the target information, before the actual search; working memory is in charge of holding the object’s sensory features relevant to the search task, that are not directly accessible via the senses. Guidance, on the other hand, refers to the process of gathering target-related information in parallel, while selection refers to the assignment of resources to the potential targets, and identification, to the process of binding the object’s features for the subsequent recognition of the target’s identity. The color associated with a flavor may therefore be represented in working memory while searching for a specific flavor label, and potentially, those flavor labels that are presented in a manner that is consistent with the statistical regularities of the environment (see Spence, 2011 and Parise and Spence, 2013), such as cucumber in green, may be easier to classify and hence to search for (cf. Vickery et al., 2005). Importantly, search efficiency is affected by both congruence and the strength of the association. This result is particularly intriguing given the existing evidence suggesting that search is guided by preattentive features such as color, as suggested by Eimer (2014; see also Wolfe, 2007). There is, however, research suggesting that when a mismatch is found (e.g., an incongruent color after a flavor cue) a deeper search is performed, which could influence RTs (Grossberg et al., 1994). Furthermore, as the color of the distractors could also match participants’ color representation of a flavor, search may have been less efficient.

It will be interesting in future research to look at how color/flavor associations affect a person’s search for a particular product flavor (i.e., would red be associated with tomato in a yogurt, say?). Another interesting direction for future research concerns target/distractor similarity, since that has been shown to influence search times (e.g., Duncan and Humphreys, 1989). In effect, products are generally presented in supermarket shelves, grouped by brands and thus by their visual appearance. Since the position of the target and distractors was always randomized and the distractors were randomly selected from a pool of distractors, some trials included two or more packages with the same color; thus it is difficult to say whether target distractor similarity exerted any influence on the search process. Anyhow, the supermarket aisle can be thought of as representing a highly complex visual search environment, in which color/flavor associations may play a key role.

In summary, we provide evidence for the idea that the congruence between color and flavor is a key element when searching for flavor information. We believe that the task used here could be fruitful for both researchers and marketing practitioners. For the former, this type of task may help to assess the influence of crossmodal associations captured in language (e.g., colors and flavor words) on search efficiency. For the latter, it may provide useful when assessing which color to use when, for example, a new product is launched.

## Conflict of Interest Statement

The authors declare that the research was conducted in the absence of any commercial or financial relationships that could be construed as a potential conflict of interest.

## Footnotes

1 The data analyses reported in Experiments 1 and 2 were also performed with these data included. Note that the pattern of results was statistically similar to the results reported in the main text.
